# Antimicrobial resistance landscape in Africa: Perspectives from the World Health Organization GLASS report 2025

**DOI:** 10.1016/j.ijregi.2026.100866

**Published:** 2026-02-23

**Authors:** Emnet Tesfaye Shimber, Mikiyas G. Teferi, Kebron W. Aweke, Biruktawit Zemede Lemma, Getaw W. Hassen

**Affiliations:** 1 Department of Emergency Medicine, African Health Sciences University, Kigali, Rwanda; 2 School of Medicine, College of Health Sciences, Addis Ababa University, Addis Ababa, Ethiopia

**Keywords:** Antimicrobial resistance, Africa, WHO GLASS, Antimicrobial stewardship, Surveillance systems

## Abstract

Antimicrobial resistance (AMR) represents a growing public health threat in Africa, where surveillance capacity and antimicrobial stewardship remain limited. Using findings from the World Health Organization (WHO) Global Antimicrobial Resistance and Use Surveillance System (GLASS) Report 2025, this commentary highlights the disproportionate burden of resistant infections across the continent, major gaps in laboratory infrastructure, and incomplete national surveillance coverage. Weak diagnostic capacity, widespread empirical antibiotic use, and misalignment with WHO Access, Watch, and Reserve (AWaRe) targets further exacerbate resistance. Strengthening AMR surveillance, diagnostics, and antimicrobial stewardship, and governance across all levels of the healthcare system is essential to mitigate AMR and advance Universal Health Coverage in low-resource settings.

## Highlights

• Africa bears a disproportionate burden of antimicrobial resistance despite limited surveillance coverage.
• World Health Organization (WHO) Global Antimicrobial Resistance and Use Surveillance System data reveal major gaps in laboratory capacity and bloodstream infection surveillance across the continent.
• Antimicrobial stewardship implementation remains weak at community, district, and tertiary care levels.
• Misalignment with WHO Access, Watch, and Reserve (AWaRe) targets and widespread empirical antibiotic use accelerate resistance.

The recent World Health Organization (WHO) Global Antimicrobial Resistance and Use Surveillance System (GLASS) report analyzed data from 23 million bacteriologically confirmed infections tested across 22 antibiotics from 104 countries. The report highlighted that resistance in Africa is higher (one in five infections) compared with the global data (one in six bloodstream infections). This report included data from 27 African countries (57.4%), indicating that the true burden of antimicrobial resistance (AMR) across the continent is still not known. Compared with Europe (58.5%) and Asia (90.9%), Africa’s AMR surveillance coverage is low. The report showed a higher burden of AMR in countries with limited surveillance systems and weak healthcare systems [1].

Data from bloodstream infections show that third-generation cephalosporin resistance for Gram-negative bacteria surpasses 70%, which is significantly higher than the global level of 44.8% (95% confidence interval [CI]: 39.3–50.4) for *E. coli* and 55.2% (95% CI: 48.5–61.7) for *K. pneumoniae.*

Although AMR surveillance coverage has improved between 2016 and 2023, only 17 countries in the WHO African Region reported nationally representative bloodstream infection surveillance coverage in 2023 (**WHO GLASS Report 2025;** Supplementary Table S1). Across all infections, the median resistance in Africa was 19.6% (range, 4.2–55.4), compared with the global median of 17.2% (range, 3.5–39.5) [1].

In the GLASS systematic review of AMR surveillance literature (2018–2023), the WHO African Region contributed a relatively small share of the global evidence base. According to the WHO GLASS Report 2025 (Supplementary Table S2), African studies accounted for 9.6% of bloodstream and gastrointestinal articles and 13.0% of urinary tract infection articles. In terms of isolates, the African Region contributed 2.2% of bloodstream and gastrointestinal isolates and 8.7% of urinary tract isolates, highlighting the continued underrepresentation of the region in global AMR surveillance despite its large population [1].

Infrastructure-wise, only 1.3% of clinical laboratories are designated to provide microbiology services; among these laboratories, only 18% have access to an automated system. This substantially limits the effectiveness of AMR surveillance and control efforts [1,2].

Drawing on regional and country-level evidence, antimicrobial resistance dynamics in the African Region are shaped by context-specific structural and ecological factors [3,4]. Limited surveillance coverage and laboratory capacity continue to constrain accurate estimation of resistance patterns across many countries. In addition, inappropriate antimicrobial use in both human and livestock sectors remains a major driver of resistance, reinforcing the importance of a One Health approach. Studies across African settings have shown that misuse and overuse of antibiotics in healthcare and agriculture, combined with limited stewardship and regulatory oversight, contribute significantly to the emergence and spread of resistant pathogens [5,6]. Addressing these region-specific challenges requires strengthening surveillance systems, expanding laboratory infrastructure, and implementing coordinated antimicrobial stewardship and infection prevention strategies tailored to national contexts.

Most community members rely on self-prescribed medications for flu-like symptoms and other minor self-limited illnesses. Additionally, the absence of a robust regulatory system for pharmacies and pharmaceutical distribution facilitates the widespread use of antibiotics in the community [7,8].

Consistent with the WHO GLASS “Priorities for Action,” overcoming structural and operational barriers to data collection remains critical for the African Region [1]. Limited clinical microbiology infrastructure, particularly at district and secondary healthcare levels, continues to constrain the coverage, representativeness, and clinical utility of national AMR surveillance systems [9]. In many settings, patients receive multiple empiric antimicrobial courses before reaching tertiary facilities, reducing opportunities for culture-guided therapy and contributing to resistance selection. Strengthening laboratory networks, improving diagnostic stewardship, and expanding surveillance beyond tertiary hospitals are essential to generate timely and representative AMR data within the African context [10].

In line with GLASS recommendations for integrated intervention packages, coordinated implementation of infection prevention and control, water, sanitation, and hygiene, vaccination, antimicrobial stewardship, and laboratory strengthening is necessary to curb resistance. Across many African countries, antimicrobial stewardship programs remain limited, particularly in lower-level facilities and the private sector, while access to WHO Access, Watch, and Reserve (AWaRe) “Reserve” antibiotics is constrained by cost, supply chain, and regulatory barriers. Expanding stewardship capacity, strengthening pharmacovigilance systems, and improving access to quality-assured diagnostics and essential antimicrobials are critical to improving antimicrobial use [11].

The burden of antimicrobial resistance disproportionately affects vulnerable populations, including rural communities, children younger than 5 years, pregnant women, and populations with limited healthcare access, thereby exacerbating inequities and hindering progress toward Universal Health Coverage. Addressing these disparities requires targeted, context-specific interventions integrated within national AMR action plans [[10], [11], [12]].

Several African countries have demonstrated feasible and scalable approaches. Nigeria’s National Action Plan on AMR expanded surveillance through digital platforms, South Africa implemented pharmacist-led antimicrobial stewardship programs, and Kenya strengthened One Health surveillance linking human and animal health systems [[13], [14], [15]]. These country-level experiences highlight the importance of strengthening surveillance, expanding laboratory capacity, and implementing integrated antimicrobial stewardship and infection prevention strategies tailored to regional contexts.

Overall, strengthening surveillance systems, improving diagnostic capacity, optimizing antimicrobial use in accordance with WHO AWaRe targets, and reinforcing laboratory information and pharmacovigilance systems are central to addressing antimicrobial resistance in Africa. Sustained political commitment, financing, and data-driven policy implementation will be essential to improve AMR control and advance progress toward Universal Health Coverage across the region.

## Declaration of competing interest

The authors have no competing interests to declare.

## Appendix Supplementary materials

Image, application 1
