# Supplementary material for: Antimicrobial resistance landscape in Africa: Perspectives from the World Health Organization GLASS report 2025
Source: IJID Reg. 2026 Feb 23;19:100866. doi: 10.1016/j.ijregi.2026.100866 (PMC13018951; doi:10.1016/j.ijregi.2026.100866)
Supplement: Supplementary file 1 [file mmc1.docx]

**Supplementary Table S1 (WHO GLASS Report 2025, Table 2.2).** Trends and current AMR surveillance coverage: median annual change (2016–2023) and 2023 national coverage, by WHO region and infection type.

| **Infection type** | **WHO region** | **Trend** | **Annual % changeᵃ** | **2023 AMR surveillance coverageᵇ** | **No. of countriesᶜ** |
| --- | --- | --- | --- | --- | --- |
| **Bloodstream** | African Region | Increasing | 14.5 (5.0, 24.7) | 35.5 (25.8, 53.4) | 17 |
|  | Region of the Americas | Increasing | 18.1 (3.0, 35.8) | 64.6 (44.6, 103.3) | 7 |
|  | South-East Asia Region | Increasing | 26.8 (11.7, 43.9) | 22.1 (16.8, 31.3) | 9 |
|  | European Region | Increasing | 14.5 (6.6, 23.0) | 440.1 (384.7, 512.9) | 31 |
|  | Eastern Mediterranean Region | Increasing | 24.0 (14.9, 33.8) | 36.1 (30.3, 44.6) | 19 |
|  | Western Pacific Region | Stable | 4.8 (–2.3, 12.4) | 528.8 (394.0, 771.1) | 9 |
|  | **Global** | Increasing | 20.0 (13.4, 26.8) | 120.3 (107.3, 139.6) | 92 |
| **Gastrointestinal** | African Region | Stable | 6.5 (–4.5, 18.9) | 3.0 (2.0, 5.2) | 11 |
|  | Region of the Americas | Stable | 4.2 (–9.1, 19.6) | 14.1 (10.4, 20.4) | 6 |
|  | South-East Asia Region | Increasing | 17.4 (1.2, 36.1) | 0.5 (0.4, 0.8) | 6 |
|  | European Region | Stable | 9.9 (–2.0, 23.4) | 38.4 (31.0, 52.2) | 18 |
|  | Eastern Mediterranean Region | Increasing | 13.9 (3.6, 25.4) | 5.0 (4.1, 6.5) | 16 |
|  | Western Pacific Region | Stable | –1.8 (–9.1, 6.3) | 17.1 (12.9, 23.5) | 7 |
|  | **Global** | Increasing | 11.4 (3.0, 20.4) | 8.3 (7.2, 9.9) | 64 |
| **Urinary tract** | African Region | Increasing | 20.2 (8.2, 33.7) | 93.2 (57.5, 185.2) | 12 |
|  | Region of the Americas | Stable | 12.5 (–1.1, 28.4) | 705.9 (473.7, 1218) | 6 |
|  | South-East Asia Region | Increasing | 31.9 (15.5, 50.6) | 47.4 (35.4, 68.1) | 8 |
|  | European Region | Stable | 5.8 (–6.8, 20.4) | 10 368.1 (8063, 13 832) | 10 |
|  | Eastern Mediterranean Region | Increasing | 37.8 (27.0, 49.3) | 211.2 (176.1, 264.9) | 19 |
|  | Western Pacific Region | Stable | 5.5 (–2.4, 14.0) | 1880.3 (1360.3, 2795.4) | 7 |
|  | **Global** | Increasing | 26.0 (17.3, 35.4) | 769.5 (644.5, 943.2) | 62 |
| **Urogenital gonorrhoea** | African Region | Stable | –7.4 (–25.2, 13.4) | 2.0 (1.4, 3.3) | 6 |
|  | Region of the Americas | Stable | –8.0 (–22.5, 8.4) | 10.4 (6.8, 18.0) | 5 |
|  | South-East Asia Region | Stable | –10.3 (–34.3, 22.2) | 0.1 (0.1, 0.2) | 3 |
|  | European Region | Stable | 4.1 (–6.5, 16.0) | 9.6 (7.6, 12.8) | 18 |
|  | Eastern Mediterranean Region | Stable | 4.5 (–9.7, 20.3) | 1.2 (0.9, 1.7) | 10 |
|  | Western Pacific Region | Stable | –9.3 (–17.9, 0.5) | 18.9 (12.8, 30.9) | 6 |
|  | **Global** | Stable | –6.4 (–18.8, 7.8) | 4.8 (3.9, 6.4) | 48 |

**Footnote:** ᵃ Population-weighted median national yearly percentage change in the number of infections with AST per million population, reported globally and by WHO region. Median population-weighted national coverage from Bayesian regression models. ᶜ Number of reporting countries included the analysis (including three territories and areas).

**Source/Acknowledgement (recommended line under the table):** *Source:* World Health Organization. Global Antimicrobial Resistance Surveillance System (GLASS) Report 2025. Table 2.2. *Reproduced with acknowledgement of WHO.*

**Supplementary Table S2 (WHO GLASS Report 2025, Table 4.1).** Numbers of articles and isolates included in the systematic review, by infection type.

| **Category** | **Group** | **Bloodstream: No. of articles** | **Bloodstream: No. of isolates** | **Gastrointestinal: No. of articles** | **Gastrointestinal: No. of isolates** | **Urinary tract: No. of articles** | **Urinary tract: No. of isolates** |
| --- | --- | --- | --- | --- | --- | --- | --- |
| **Year of isolate collection** | 2018 | 120 (31.3) | 81 711 (33.6) | 128 (26.4) | 360 704 (35.9) | 11 (20.8) | 1079 (20.0) |
|  | 2019 | 117 (30.5) | 90 334 (37.2) | 133 (27.5) | 333 603 (33.2) | 20 (37.7) | 2127 (39.3) |
|  | 2020 | 71 (18.5) | 42 560 (17.5) | 94 (19.4) | 143 926 (14.3) | 13 (24.5) | 1296 (24.0) |
|  | 2021 | 50 (13.1) | 18 627 (7.7) | 73 (15.1) | 92 516 (9.2) | 5 (9.4) | 610 (11.3) |
|  | 2022 | 23 (6.0) | 9497 (3.9) | 47 (9.7) | 73 287 (7.3) | 2 (3.8) | 296 (5.5) |
|  | 2023 | 1 (0.3) | 100 (0.0) | 8 (1.7) | 1614 (0.2) | 0 (0.0) | 0 (0.0) |
| **Health-care level** | Tertiary | 244 (80.0) | 115 356 (47.5) | 310 (71.3) | 409 848 (40.8) | 28 (59.6) | 3482 (64.4) |
|  | Secondary | 20 (6.6) | 2707 (1.1) | 32 (7.4) | 8042 (0.8) | 7 (14.9) | 531 (9.8) |
|  | Primary | 3 (1.0) | 309 (0.1) | 38 (8.7) | 16 197 (1.6) | 4 (8.5) | 512 (9.5) |
|  | Unknown | 38 (12.6) | 124 457 (51.3) | 54 (12.4) | 571 563 (56.8) | 6 (12.8) | 883 (16.3) |
| **WHO region** | African Region | 30 (9.6) | 5463 (2.2) | 43 (9.6) | 22 534 (2.2) | 6 (13.0) | 468 (8.7) |
|  | Region of the Americas | 30 (9.6) | 49 871 (20.5) | 21 (4.7) | 221 338 (22.0) | 3 (6.5) | 770 (14.2) |
|  | South-East Asia Region | 70 (22.4) | 17 209 (7.1) | 94 (20.9) | 22 947 (2.3) | 6 (13.0) | 638 (11.8) |
|  | European Region | 48 (15.4) | 35 530 (14.6) | 77 (17.1) | 379 376 (37.7) | 2 (4.3) | 107 (2.0) |
|  | Eastern Mediterranean Region | 53 (17.0) | 22 067 (9.1) | 188 (41.9) | 46 335 (4.6) | 16 (34.8) | 2330 (43.1) |
|  | Western Pacific Region | 80 (25.6) | 112 689 (46.4) | 25 (5.6) | 313 120 (31.1) | 12 (26.1) | 1095 (20.2) |
|  | **Total** | **305** | **242 829** | **45** | **5 408** | **434** | **1 005 650** |

ᵃ The proportion of the total number of articles and isolates in different years, by health-care level and by WHO region is given in parentheses. Some articles are counted in more than one category. *Source:* World Health Organization. Global Antimicrobial Resistance Surveillance System (GLASS) Report 2025. Table 4.1. *Reproduced with acknowledgement of WHO.*
